# “Dying a hero”: parents’ and young people’s discourses on concurrent sexual partnerships in rural Tanzania

**DOI:** 10.1186/1471-2458-14-742

**Published:** 2014-07-22

**Authors:** Joyce Wamoyi, Daniel Wight

**Affiliations:** 1 Department of Sexual and Reproductive Health, National Institute for Medical Research, P.O Box 1462, Mwanza, Tanzania; 2 Medical Research Council/CSO Social and Public Health Sciences Unit, University of Glasgow, 200 Renfield St, Glasgow G2 3QB, UK

**Keywords:** Concurrent sexual partnerships, Discourses, Tanzania, HIV/AIDS, Young people

## Abstract

**Background:**

Concurrent sexual partnerships (CSPs) have been speculated to drive the HIV pandemic in many sub-Saharan African countries. We have limited understanding of how people think and talk about CSPs, how beliefs are transmitted across generations, and how this might affect the practice. This paper explores these issues to understand how CSPs are perpetuated and help identify opportunities for interventions to modify them.

**Methods:**

The study employed an ethnographic research design involving: participant observation in 10 households, 60 in-depth interviews (IDIs), and nine participatory focus group discussions (FGDs). Participants were young people aged 14-24 and parents/carers of young people within this age group. The 60 IDIs were conducted with: 17 fathers, 13 mothers, 13 young men and 17 young women (six of whom had had unplanned pregnancies and 11 had no children). The nine FGDs were conducted with groups of: fathers (2), mothers (2), young women (2), and young men (3). A discourse analysis was carried out with all the transcripts. Data were analysed with the aid of NVIVO 8 software.

**Results:**

Six distinct discourses were identified from the way participants talked about CSPs and the norms driving the practice: 1) predatory masculine sexuality; 2) masculine respectability; 3) feminine respectability; 4) empowered modern women; 5) traditional health beliefs; 6) public health. Discourses legitimating CSPs were drawn on and reproduced primarily by young people and the media and only indirectly by parents. Discourses discouraging CSPs were used primarily by parents, religious leaders and learning institutions and only indirectly by young people themselves.

**Conclusion:**

Better knowledge of the discourses through which young people CSPs, and how these discourses are transmitted across generations, might help develop “culturally compelling” interventions that modify these discourses to enhance sexual health.

## Background

Despite decades of public health interventions in sub-Saharan Africa, sexually transmitted infections (STIs) and HIV remain serious health problems among young people. The limited impact from rigorously evaluated behavioural interventions [1,2] suggests the importance of entrenched social factors shaping sexual behaviour [3]. Previous sexual health interventions have emphasised the importance of changing adolescents’ proclivity to take risks (especially their knowledge and attitudes), but robust evaluations have not shown any long-term behaviour change or improved sexual health outcomes at the population level [2]. There is now a growing interest in how the broader social environment puts people at risk and hence the focus on structural approaches to improving adolescent sexual health [4,5].

Previous research overwhelmingly supports the notion that parents influence their children’s decisions regarding sexual behaviour [6-10]. Yet very limited attention has been given to understanding the socialization of children and how social and sexual norms are transmitted across generations. Dilger [11] argues that conceptions of socialization have been rendered ambiguous by globalization, modernity and AIDS messages. The values that are imparted to young people from family or peers often conflict with the preventive advice provided from other sources. In order to make adolescent HIV/AIDS prevention more effective it would help to take into account the perspectives of young people and those who socialise them, for example by engaging with how people talk about their sexuality.

The term concurrent sexual partnerships (CSPs) is used to describe a situation where sexual partnerships overlap in time, either where two or more partnerships continue over the same time period or where one partnership begins before the other ends [12]. While considerable debate remains over the significance of concurrency in the HIV epidemic [13-18], several authors have speculated that high levels of multiple and CSPs coupled with insufficient, inconsistent condom use are driving the HIV pandemic in many sub-Saharan African countries [1,19-21]. Surveys rarely investigate CSPs given the difficulties in defining them. The survey data available in Tanzania concern multiple partnerships which might be serial or concurrent. In the recent Tanzania Health and Malaria indicator survey, the proportion of 15-49 year olds who reported they had had two or more sexual partners in the past 12 months was 4 percent for women and 21 percent for men [22]. In another report, the Tanzania commission for AIDS found that younger women were more likely than older women to report multiple partners, whereas older men were more likely than younger men to engage in multiple partnerships [23] (TACAIDS, et al.). It has been suggested that the only long term sustainable solution to the present HIV/AIDS crisis in sub-Saharan Africa is a change in sexual norms and behaviour [3,24]. However, we have limited understanding of how people think about and discuss CSPs, how beliefs are transmitted across generations, and how this might affect the practice of CSPs.

Discourses involve more than language: they organise meaning and action [25]. In our analysis, we use discourses in Hollway’s [25] sense to mean a cohesive set of spoken ideas that are shared socially but not necessarily held in their entirety by any one individual. Discourses are constitutive of the social world that is a focus of interest or concern. They reflect how people think about topics and probably shape their thinking. Dominant discourses produce and govern the ways in which knowledge about sexual health (in our case CSPs) can (and cannot) be discussed [26]. It has been noted that discourses promote particular kinds of behaviour [27], although the extent to which discourse and practice actually match-up may be difficult to ascertain and is beyond the scope of this paper. In her analysis of heterosexual relationships, Hollway [25] argues that people’s roles arise from the way they position themselves within particular discourses related to sex. Hence, by exploring young people’s discourses about CSPs we can better understand how their discussions of sexual relationships are discursively constituted, constructed and changed [28].

To date there is little empirical or theoretical literature examining discourses on CSPs, in particular young people’s discussions with their peers regarding this practice and parental views on CSPs, especially with regards to their children’s involvement in the practice. This analysis is part of a larger study that set out to investigate how beliefs and practices concerning sexual relationships are transmitted across generations. Here we explore parental and young people’s discourses used to discuss CSPs, how they are transmitted across generations, and how this might affect CSPs practice. A better understanding of these processes might help tailor sexual health interventions intended to modify the cultural context of CSPs [29].

## Methods

The study took place in Magu District, Mwanza Region in northern Tanzania, amongst a rural and peri-urban population who were predominantly Sukuma. The study participants were young people aged 14-24 years and parents with children within this age-group.

Ethical approval for the study was provided by the Tanzanian Medical Research Co-ordination Committee. Additional permission to conduct the study was granted at district, ward, and village levels. In addition to seeking the consent of participants, for those aged below 18 years (the age of majority in Tanzania), consent was also sought from parents or caregivers. The purpose of, and methods for, the study were explained to potential participants, who provided verbal consent prior to participating. The design, implementation and reporting of study findings complies with the relevance, appropriateness, transparency of procedure and soundness of interpretive approach (RATS) guidelines for qualitative research.

### Design

This study employed an ethnographic research design. Data were collected using participant observation (PO), in-depth interviews (IDIs), and focus group discussions (FGDs). Combining these methods increased our understanding of complex issues related to how parents and young people talked about CSPs. As noted by several authors [30], the best way to understand family interactions and the experience of family membership is by observing and interacting with families. The PO collected data on familial interactions, child socialisation and transmission of sexual norms and behaviours across generations. The FGDs focused on how participants collectively made sense of CSPs and young people’s sexual behaviour, and the attitudes and values they were prepared to disclose with their peers. IDIs allowed parents and young people to reflect individually on their understanding of CSPs, how they thought and talked about them, and for the young people their personal experiences of CSPs.

### Data generation

Data were collected in two phases by three graduate researchers (first author included). Two were from the Sukuma ethnic group like the majority of the participants. The first phase involved PO, nine participatory FGDs and 29 IDIs, while Phase 2 involved 31 IDIs plus two repeat interviews with young women interviewed in Phase 1.

Sampling for PO included villages, families and young people and was done with the help of the village authorities. Ten households/families were selected from one village for PO, initially on the basis of convenience sampling, recruiting people the researchers met and using their social networks to recruit others (snow ball sampling). Subsequently purposive sampling was employed to ensure that the ten households/families were representative of the different household types in the community: six dual-parent households (4 non-polygamous and 2 polygamous) and four single-parent households (1 single father, 3 single mothers). The ten households were selected from about 150 households.

One of two researchers (1 male, 1 female: JW) spent three days in each of the selected households, getting to know the families, trying to establish a trusting rapport with them, and observing the young people’s familial environment. Observing naturally occurring parent-adolescent interactions was likely to provide more valid data than interview or FGD accounts, and allowed us to triangulate the IDI and FGD data. It was not intended to collect data on sexual behaviour.

A checklist was used to focus observations, including: family socio-economic status, parental presence and interactions with children, time parents spent with their children, and references to sexual relationships, young people's behaviour and unplanned pregnancies. Some of these themes were trigger points for discussions in the IDIs with some of the participants. Jottings were taken in the course of the day and detailed notes written up at the end of each day describing important observations. The two researchers met daily to review progress and their focus.

At the end of PO, FGDs and IDIs were conducted with some of the participants from the PO village and from six other villages within the same ward. Ninety people (20 fathers, 20 mothers, 20 young women and 30 young men) participated in nine FGDs, each comprised of 10 participants and conducted by a researcher of the same sex. Both purposive and snowball sampling were used for the selection of participants. With the help of the Magu demographic surveillance site field officers, initial participants (especially parents) were identified from different family types (single father, single mother, two parents) that had emerged as important for young people’s upbringing during participant observation. Three days were then spent getting to know and recruiting the pre-existing friendship groups of these initial contacts, so that they knew each other well and were free to discuss sensitive issues in each other’s presence [31]. The FGDs with parents were organized according to gender, while those with young people were by gender and schooling status (in and out-of-school).

A total of 60 IDIs were conducted with: 17 fathers, 13 mothers, 13 young men and 17 young women (6 of whom had had unplanned pregnancies and 11 had no children). With 10 of the young people interviewed others in the same family were also interviewed: both parents (3 families), one parent (2 families) and a brother or sister (5 families). This enabled a more detailed understanding of familial interactions from the perspectives of different family members and the triangulation of responses at a family level. The remaining interviews did not involve people from the same families.

The IDIs were held with FGD participants so as to build on the rapport established during the group discussion and to explore at a personal level some of the issues that had emerged. Interviewees were purposively sampled to represent different family types, to explore experiences of unplanned pregnancy as young mothers or grandparents, and to include both dominant and reserved group discussion participants. Initially, 29 IDIs were conducted. Preliminary analysis of the Phase 1 data identified remaining gaps in our knowledge and new issues that required exploration. This was done in Phase 2 using an additional 31 IDIs with people selected through theoretical sampling [32]. The new issues explored in additional IDIs were: understanding of CSPs versus serial monogamous partnerships, norms supporting CSPs, norms discouraging CSPs and how norms on CSPs were transmitted across generations. Two of the young women interviewed in Phase 1 who had had unplanned pregnancies were interviewed again in Phase 2 in order to follow-up on central issues. The research was conducted in Swahili, the national language of Tanzania.

Both the FGDs and IDIs were participatory in nature, involving activities that helped participants differentiate between CSPs and serial monogamy. Such activities involved drawing symbols to represent different partners and then working through what we meant by CSPs. This resulted in drawing networks of partners with arrows pointing back and forth to represent overlap in partners at particular time points. The researchers further clarified what they meant by CSPs by explaining that a CSP entailed an individual having sex with one partner on more than one occasion (e.g. over a period ranging from two days to six months) and during this period having sex with someone else. These exercises helped participants to think through the practice of an individual having sex with different people in an overlapping manner, but also conceptualise the risks in CSPs.

### Analysis

Following the two phases of data collection, tapes were transcribed verbatim and a random sample translated from Swahili into English for the non-Swahili speaking senior co-investigator to confirm the emerging themes and provide feedback. All the data collected during Phase 1 and 2 were entered in NVIVO 8 software for coding. A coding framework was developed in two main stages. Initially the two authors used a random selection of IDI and FGD transcripts and PO notes from Phase 1 to develop six broad codes that were used to code five FGDs and seven IDIs plus the observation notes. These codes were both a priori as well as grounded in the data and were developed in close consultation with the two other graduate fieldworkers. In the second stage the coding frame was revised in the light of Phase 2 data and to develop finer codes, again in discussion with the whole research team. All the remaining data and that collected during Phase 2 were then coded according to the revised coding frame.

The two authors then thoroughly examined the coded data for emerging patterns. For example, the connection between concepts such as CSPs and serial monogamy, and examined how participants talked about CSPs when with peers, sexual partners and children. The analysis assumed that young people were both influenced by discourses and engaged in shaping and reproducing them. The transcripts were analysed to explore subverting and contesting discourses by asking: what dominant discourses do young people employ when talking about CSPs with peers, adults and sexual partners, what dominant discourses do parents employ when talking about CSPs with their children and their peers, and what are the specific ways that young people either appropriate and construct, or resist and challenge, dominant CSPs discourses? Theories were formulated, such as: ‘the way parents and young people talked about CSPs was encouraging the practice’. In order to test this theory, ‘child codes’ and ‘parent codes’ relating to parents’ and young people’s views on CSPs, CSPs and assessment of risk, and transmission of discourses on CSPs were searched, summarised and compared. We tried to use all the relevant data to test initial theories, many of which were then rejected or modified.

Quotations illustrating the main findings were identified. In the presentation of the quotes, ‘I’ refers to the interviewer while ‘R’ is the respondent.

## Results

### Socio-demographic characteristics of participants

The participants were young people aged 15-24 years (median age 20) and parents of young people within this age group. The parents were aged 35-65 years (median age 53). The main means of livelihood for most parents was subsistence farming. A few engaged in income earning activities through petty trade within their villages and surrounding areas. The majority described themselves as Christians.

Ten of the young women interviewed lived with both parents while the remaining lived with single mothers. For the young men, 4/13 lived with single mothers while the remaining nine lived with both parents. Six of the young women who participated in FGDs and IDIs were unmarried mothers having had a teenage pregnancy. Four of the parents (3 mothers, 1 father) reported that they were single as a result of the death of their spouse.

All but four of the young people had completed primary schooling, while half the parents had not. Seven of the young men and eight of the women had some secondary school education although none had been successful to continue with higher levels of secondary school.

### Context of young people’s sexual relationships

In six out of the seventeen IDIs with young women, they reported that although they had been approached by several men to establish sexual relationships, they had never had sex. Two of these women were in secondary education while the remaining were in primary school. Both parents and young people talked about abstinence until marriage as the ideal behaviour, and if one could not abstain one should have one sexual partner. However, they reported that CSPs were the commonest type of sexual partnerships among the unmarried young people in their communities and many believed that every one engaged in the practice:

No one has one lover, maybe there are, but these would be very few. (IDI, out-of-school young woman)

Six out of the 11 sexually active young women reported they had engaged in CSPs at some point of their relationships. For the young men, only one said he was not sexually active. The rest were sexually active and had at some point of their relationships engaged in CSPs. Out-of-school and secondary school young women were said to be more likely to have CSPs than primary school girls.

R1: You’d find she has a number of them

I: And who are those with many partners?

R2: Even primary school [children] do it…but the primary school ones are few …Mostly it is those ones in secondary school and colleges that mix a lot. (FGD, out-of-school young women)

Four out of the six out-of-school young women who had had unplanned pregnancies, reported that they had engaged in CSPs in order to meet their material demands and those of their babies.

Young people’s sexual relationships can be characterised as moving from serial monogamy to CSPs. Some sexual encounters with certain partners were episodic, especially if the partner lived away from their village. In such situations, young people reported they had another sexual partner when the other one was away and when s/he returned, resumed sexual encounters with these partners as they were perceived as long term. Among many factors that determined young men’s decision to engage in CSPs were: establishing masculine identity, peer influence, access to money and ability to pay for sexual exchange. For the young women, these factors were: material demands, previous experience of unplanned pregnancy and peer influence. These factors were expressed and reinforced through discourses.

From the analysis of these young people’s and parents’ accounts, we delineate six discourses which we refer to as: predatory masculine sexuality, masculine respectability, feminine respectability, empowered modern women, traditional health beliefs, and public health. The findings are structured according to these discourses. In discussing these discourses, we reflect on the role of peers and parents as socialisation agents in shaping some of these discourses.

### Discourse of predatory masculine sexuality

This discourse expressed the stereotype of masculine sexuality in which men gain esteem from their male peers through seducing as many women as possible to become sexual partners. Men felt a sense of pride in discussing high numbers of partners and CSPs as an expression of their physical strength and superior seduction skills. In this discourse there seemed to be little value in maintaining a relationship with a partner once one had already had sex with her. As a result, the predatory masculinity discourse encouraged short term relationships with minimal commitment on the man’s side. Mothers mentioned that they were instrumental in socialising their children to understand the double standards of masculinity e.g. “men are always men and they are different from women”.

Young men and women discussed how different women performed differently during sex. They talked about some women’s sexual performance being better than others and hence men desired to experiment in having sex with as many women as possible. This feeling was important in promoting CSPs and encouraging casual partnerships. Young women said they were aware that men shared their sexual experiences with peers:

For males, their only benefit is that “I have got her”, I have got so and so and I have passed through her…Yea, they are so proud when they say that …Also when a man sits with his peers once you pass, he starts saying “this girl is great at making love”. Ee, they will be happy to hear that, even others will approach you [woman] to prove what they have heard. (IDI, out-of-school young woman)

Men were considered the “approachers” (expected to seduce a woman) and “providers” (through transactional sex) in sexual relationship and hence they had the liberty to do this with as many women as they could convince. The discussions among peers that men are the “approachers” and “providers” while the women are the “approached” and the receivers encouraged predatory behaviour among males. A young man talked about his experience:

I am a DJ [disco jockey] and that makes one attractive in the village…You just cheat them [women]…When I have my ear phones on and a girl requests for a song, I play it for her but also call her to come and listen to it through my ear phones and that is when I get an opportunity to talk [seduce]. You know when you are a DJ, it is like you are selling your looks…through that work I have three [partners] in addition to a fourth one…You know in doing that work you have some pride, mainly through cash. You know when you have cash in your pocket, nothing is impossible. Everything is simple and you can desire any woman and you just send your friend to check her out for you. (IDI, out-of-school young man)

Fathers and young men reported how their peers talked about having CSPs as an expression of a man’s physical strength (energy) which is an important male virtue but also a sign of male sexual superiority and superior seduction skills. A father said:

Since he wants to show his peers that he is able, he is good, he knows how to talk or he is strong, that is what brings the competition (IDI, father, two parent family)

### Discourse of masculine respectability

The masculine respectability discourse stressed that men’s respectability partly emanates from their provision for their families and sexual partners. Participants talked about the traditional male provider role and how, as with polygamy, CSPs demonstrated a man’s ability to provide for the women and large families which was indicative of his wealth.

It is likely that the manifestation of a man’s wealth through providing for many wives has now shifted to providing for many partners through CSPs. Having CSPs was perceived as expensive and a man’s engagement in the practice was a sign of wealth since he could afford sexual exchange with many partners:

I mean for a person who is poor, he cannot manage/handle relationships with many lovers…those who look for many lovers are those with money. (FGD, in-school young men)

While young women talked about the financial benefits of having CSPs, it was interesting to note that mothers, fathers and sons discussed the costs to men of the practice. It could be the cause of poverty for a man and his family, and thus impact on the man’s respectability in a different way. A young man talked about the consequence of spending one's resources on women in the following:

Because having many partners, I mean you won’t be able to grow financially…I mean…all those women will drain you and then leave you. (IDI, out-of-school young man)

The male respectability discourse sometimes conflicted with the discourse of predatory masculinity. It was observed that men positioned themselves differently in the two discourses depending on their audience and situation. For example, when with parents, men were more likely to draw on the respectability discourse, but when with peers, young men positioned themselves more in the predatory masculinity discourse. However, the male respectability discourse could both legitimate and condemn multiple sexual partners. While on the other hand men were expected to show their wealth through having many women, a man with too many sexual partners simultaneously could lose respect in his community:

You find that people see you with different women… when you pass by, people say “that he is just only able to seduce women”. That respect for you is not there. (IDI, out-of-school young man)

This was particularly the case if he did not provide for his family:

Usually for a married man who does not provide for his wife and family but provides for a mistress, people will talk about him negatively. (FGD, out-of-school young men)

Both parents and young women discussed how intergenerational sex involving adult men with young women (usually teenage girls) reduced men’s sexual respectability. This was particularly so if the man was old enough to be considered the girl’s father. Hence men who had sexual relationships with young women were referred to using the derogatory term “*fataki”* which literally refers to explosives. The term *fataki* stigmatised men who tried to seduce young women:

When asked OE [father] the sexual behaviour he dislikes most in his community, he said that he disliked men who desired other people’s daughters as their sexual partners. He said that the behaviour of older men sleeping with small girls is common in their village, and some men even make the girls pregnant and end up with a conflict with the girls’ fathers. (PO notes, father from a polygamous family)

Fathers talked about how older men handled CSPs in a secretive manner to maintain their respect in their community:

An adult man like myself would ensure that if I wanted a *hawara* [girlfriend/partner] in addition to my wife, I would find her from outside my village and not from within where everyone knows me. (IDI, father from two parent-family)

### Discourses of feminine respectability

The feminine respectability discourse emphasised stereotypical respectable qualities as being: sexual restraint, marriageability, attractiveness to men and accepting their polygamous nature. This discourse encouraged marriage as a core goal for young women and those behaviours that made them more eligible, thus it generally discouraged CSPs. However, one aspect of the discourse that encouraged concurrency was the expectation that women should tolerate men’s polygamous nature. From men’s perspective, the discourse’s emphasis on women’s abstinence and sexual restraint discouraged CSPs.

While the discourse among most fathers was that daughters should abstain until marriage, mothers were not as clear in their communication about this. Mothers said that they talked to their daughters about the need to preserve their respect by not having CSPs:

It is required that if you are a girl maybe you just get one lover. It does not bring a good impression to have several…that is disrespectful. (IDI, mother two parent family)

In addition, mothers warned daughters that engaging in CSPs would make it difficult to find someone to marry them and referred to women who had CSPs as “changing partners as if they were changing clothes”. A young woman described her conversation with her mother:

R: She can tell you that if you have got a partner, then you should not be with a different one today, another one tomorrow, it is not good…if you decide to have a partner just have one…don’t get used to mixing them, “those are not clothes to keep changing”

I: Why was mum advising you?

R: According to her, since we are girls you can start running here and there mixing them and you lose your respect. (IDI, out-of-school young woman)

A component of this discourse that resonated among mothers and young women was that women should tolerate unfaithful partners, thus facilitating CSP among men. They believed that there were few faithful men and tolerating unfaithful partners was a necessary virtue for women to be married or sustain a relationship long term. Mothers reflected on their upbringing and discussed being socialised by their parents especially their mothers to tolerate the polygamous nature of men and that terminating one’s marriage on the grounds of unfaithfulness was very much frowned upon and would reduce a woman’s feminine respectability. Mothers talked to their daughters about the societal expectation of good behaviour for men and women:

A man can have even five partners, even four, for example if he does not want to marry all, he can have three *hawaras [lovers]* outside [extramarital] in addition to you [his wife] and you just stay with him. (IDI, mother two parent-family)

While men and women discussed the society’s tolerance of males having CSPs, women who were involved in CSPs were stigmatised with labels such as “*malaya*” (prostitute) or “*jamvi la wageni*” (literally ‘a visitors’ mat’). The association between women having CSPs with prostitution made some women conceal their sexual relationships so as to avoid such negative labelling which clearly had implications for their reputation and that of their family. Condemnation of women who engaged in CSPs seemed to discourage the practice among women. A young woman referred to how women with several sexual partners risked isolation:

For a village woman, having many partners [concurrency]…I mean you can even be isolated, whenever you pass, they will call you a prostitute…she has slept with this one and that one… I mean she is like a “*jamvi la wageni*” [visitor’s mat], every man sits on it…you know at home whoever comes will sit on it, if another one comes tomorrow he will sit just like that… but for a man he is praised. (IDI, out-of-school young woman)

Young men also talked about the differences between a respectable woman and one reputed to have several partners:

R1: Their reputation differs…the one with one partner will be respected in the community…but the one having many partners would be talked about badly.

R2: The difference is that the one without a lover is liked by the community…but the other one with different men, the community hates her. Therefore, the difference is that the community likes one without lovers [abstains] and hates the one with many partners. (FGD, out-of-school young men)

Both parents and young people talked about the expectation that women should wait to be approached by men to start a sexual relationship, and hence a woman first approaching a man was disdainful. The reputation of the approached woman was also tarnished if she readily accepted proposals for sexual relationships with different men.

I: Among the Sukuma how do people talk about a girl with many partners at the same time?

R: Frankly it is a bad reputation,

I: What about for the man?

R: For the man it is not really bad because he is the seeker, while you are the girl to be sought out, second, a woman’s value on the market usually ends and she becomes market less. She lacks respect but the man, he is allowed, even to have ten women, but the woman cannot. (IDI, mother two parent-family)

The examples above illustrate the sexual double standard in relation to CSPs, with women condemned and men sometimes praised.

### Discourse of empowered modern women

The discourse of empowered modern women celebrated the modern, trendy lifestyle adored by young women, in which they are autonomous and often entrepreneurial. It was often opposed to traditional notions of respectability as reflected in the feminine respectability discourse and was dominant among young women. It positioned women as able to exercise agency and make their own decisions in response to the socio-economic changes around them. This discourse depicted women engaging in CSPs as modern and trendy: in it the woman is the subject while the male is the object.

Both parents and young people talked about the general feelings among young people that having CSPs was being trendy/cool (“*kisasa*”). They termed this as “*kuenda na wakati*”, literally meaning “to go/move/change with the times”. When with their peers young women discussed having one partner as “*ushamba*” [backwardness/old fashioned] since he would not meet the demands of a trendy lifestyle. Young women gave examples of how they had learnt from their peers that one of the ways of showing that one had “changed with the times” [modernity] was through the number of partners one had at a time:

If you have one partner they call you *mshamba*…that to have one boyfriend is just but *ushamba* [backwardness]. (IDI, out-of-school young woman)

Young women also discussed having CSPs as a sign of bravery and cleverness:

Ee, nowadays they call it being clever… That is what they tell each other…I mean having more than one boyfriend is cleverness/cunningness. (FGD, out-of-school young women)

Here they were referring to how much they could reap from several men through transactional sex.

Young women believed that having several partners was the gateway to achieving their desires for physical/material needs, such as modern clothes and mobile phones, and keeping up with a trendy lifestyle that could not easily be fulfilled by having one partner. Different partners were perceived as playing different roles in a young woman’s life.

R: It is a must for me to have a ‘side one’ [additional partner to the main one], nowadays they say, “it is a must to have a small dish [partner who caters for small needs], probably a large trough [partner who caters for big needs]…A large trough, in addition a medium size bucket [a partner who caters for average needs], a small gallon, and a *dasani* [small bottle used for bottling drinking water”] [a partner who caters for minor needs]

I: Mm.

R: You see, that is why

I: Who says all that?

R: It is just fellow women at the market. If you speak out [against], they tell you, “no wonder you stay with one man as if he is your father”. (IDI, out-of-school young woman)

Intergenerational sex was perceived to be common among young women. Sugar daddies, locally known as *Fatakis,* were reputed as popular for affording transactional sex:

R1: Yeah … now of course if I’d tell a fellow, “Nowadays I have another cool *fataki* and you have decided to hang on to the same guy … you’ll suffer”

R2: now I’m just having good time … if so and so doesn’t have money, I go to that … um … *fataki* … how he’d give loads of money. (FGD, out-of-school young women)

It was also interesting to note that young women dismissed a faithful relationship with a single sexual partner as being like one’s relationship with one’s father, implying equivalent subjection to male authority and lack of choice:

R1: You sit down with your peers and they advise you, “Now you hang on to this one person [partner], did you descend with him from heaven? Look for others too.

R2: Now there were others who said, “I cannot have one boyfriend. Is he my father?”

I: Who was saying that?

R3: Female students

R1: You shouldn’t regard him [sexual partner] like your father … that is, you stick to him as if he is your parent [Because you can only have one father] … somehow you ought to go outside [of that relationship].

R4: The permanent one…As if he [God] has created for you something special [one partner]

R3: Ahaa … they ask … did he create him for you?

R5: Or did you descend with him [one partner] from heaven holding arms that’s why you stick on to this one person only. (FGD, out-of-school young women)

Such popular beliefs among young women clearly put them at sexual risk.

A further incentive for CSPs was uncertainty about the possibility of one’s sexual partner leaving. Having other relationships provided some sort of insurance against being left without a boyfriend:

I mean you are sticking to him [one partner] as if he is your father…On the day when he leaves you, where will you run to. (IDI, out-of-school young woman)

In both the GDs with young women, and in several IDIs, they talked about how their peers discussed the idea of having CSPs and “dying from AIDS as equivalent to dying a hero”. They said that for young people and adults who engage in CSPs it meant one was a “fighter” since they had used their existence in the world well by enjoying themselves.

I: Mm … I see … and for instance, if women or girls like you have more than one partner, what do people think of that… fellow girls for example?

R1: Yeah, nowadays that is bravery.

I: Well, they call it bravery/smartness.

R2: Mm … you know, it means that if you died of AIDS today, it would be said you were…you were a brave person, you were a fighter

I: Oh, why do they say so?

R3: Mm…Because you were a brave person…Neema (pseudonym) was a brave person…she’d fought really hard

R4: Mm, because God brought her here, and she used herself accordingly…Accordingly … I mean her gender [being female], she used it accordingly until it reached a point of contracting AIDS… she’s died like a hero indeed. (FGD, out-of-school young women)

The discourse of empowered modern women directly contradicted that of feminine respectability, and men drew on the latter discourse in their criticism of young women. They talked about women’s greed for money as one of the reasons for engaging in CSPs. Both fathers and young men said that most women expected large material favours from their sexual partners and did not tolerate men who could not provide. They instead moved to other partners so as to earn more, which impacted on their feminine respectability. Young women acknowledged their material motivation and the consequences for respectability:

It is greed for money…Money puts you at risk … you’d think, “this person gives me little … maybe I should look for someone else” … the one you’ll get also gives you little … again you’d think you should add someone else… and you just become nothing [treated with contempt]. (FGD, out-of-school young women)

### Discourse of traditional health beliefs

Research participants sometimes drew on a discourse of traditional health beliefs which discouraged CSPs and was in conflict with the discourses of predatory masculine sexuality and empowered modern women. Although not supported by bio-medicine, these traditional beliefs potentially had important public health implications.

A dominant belief held among young men and fathers was that engaging in CSPs would cause bad health due to too much sex in an attempt to serve many sexual partners. This caused fear and discouraged CSPs among men. Fathers talked about too much sex overworking one’s muscles resulting in a disfigured back and other bodily ailments.

It is very risky [CSPs]. You find that every time the muscles do something they expand…For most of the men who played that game [CSPs] during their youthhood, they have problems. You come to the women, you find that they have a bent back, because she used to do it [sex] all the time. Now that act of lifting the weight of many men, she walks while bent as if she has a broken back. (IDI, father single (widowed))

Fathers and young men also talked about too much sex leading to weight loss. Young men described the loss of weight *(kukonda*) as resulting from women “sucking” the man through frequent ejaculation, and a father explained:

“You don’t have any peace and even health wise apart from diseases everyday you give away blood [sperms] at the end of it you’ll become weak…and when you produce lots of sperms your health deteriorates”. (IDI, father in two parent family)

As indicated in the above excerpt, it was interesting to see that participants also considered psychological health as important when they talked about “lack of peace” as a result of engaging in CSPs.

Mothers and young women talked about how their communities discouraged CSPs among breastfeeding mothers by associating the practice with ill health in the child. Unmarried young women with children said their mothers had warned them against engaging in CSPs for the same reason. A young woman said:

When I was pregnant mother told me that I should not mix men “you should only have one”. Also when I gave birth she said…“you shouldn’t have a relationship with a man at all, you will make your child to lose weight if you mix men”. (IDI, out-of-school young woman)

### Public health discourse

The five discourses outlined above were all largely indigenous, in that they represented local ways of interpreting the social world, albeit often in response to global trends and, therefore, often reflecting globally dominant discourses. However, research participants also drew on understandings of HIV and sexual and reproductive health (SRH) risks that came from the public health discourse of health professionals. This discourse was dominant among young people, especially those who had attended secondary school, and no one contested it.

The one with many lovers will just die of diseases. (FGD, out-of-school young men)

Participants’ discussion of the health risks of CSPs indicated good knowledge. Most contrasted the HIV risks inherent in serial monogamy and CSPs and concluded that the latter was riskier. They discussed how HIV spreads fast in an environment where many people are engaged in CSPs versus where they are having sex with one individual and not going back. It is notable that almost all participants who reported they had had CSPs were also well aware of the risks of HIV. Parents sometimes discussed with their children the risks of engaging in CSPs and young people reflected on the warnings from parents. A young man who reported having four sexual partners also talked about how bad it was to have CSPs:

The danger is mainly, let’s say is sleeping with someone and after you go [leave him] you return again…Because you have gone up to number one, you go up to number ten, then you return again to number one, in number one you don’t know in which number s/he has entered again…But this one, the one who comes from number one and s/he does not return completely to number one but proceeds to a new one…Maybe let us say that she is at least okay because s/he doesn’t return again to bring the effects, if s/he has got them from number two s/he will take them to number three…You see because that rotation becomes very big and is different from this one who is going back and forth compared to this one who is going directly in front… yes, s/he leaves him/her completely, because it is dangerous (IDI, out-of-school young man)

Similarly, young women talked about CSPs leading to infection with HIV and other STIs.

Because you can get diseases…like syphilis and HIV. Yes, if you mix men…you find that one of them will have diseases, when you sleep with him then you go and sleep with someone else, you go to infect the other with that disease and that continues. (IDI, out-of-school young woman)

### Transmission of discourses on concurrency

**Figure 1:**
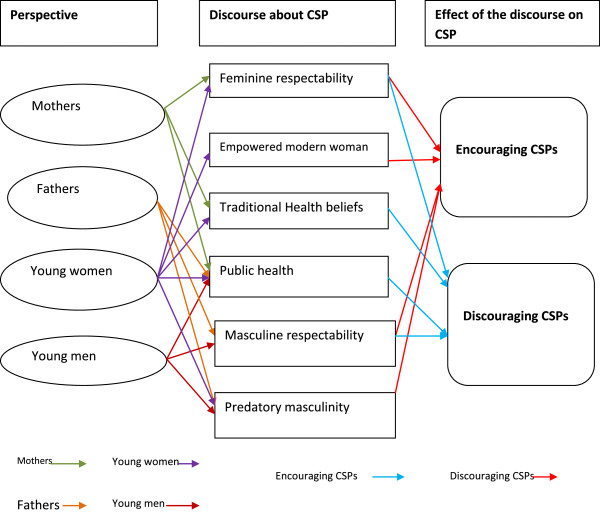
Complexities in the discourses.

The discourses were interrelated in many ways and individuals drew on them differently according to their social context. For example, young women with their peers tended to draw a lot on the discourse of the empowered modern woman, but with adults, and particularly their parents, they were more likely to draw on the feminine respectability, public health and traditional health beliefs discourses. On the other hand, young men drew a lot on the predatory masculinity discourse while discussing CSPs with peers, while with adults they were more likely to draw on the public health and men's respectability discourses. Hence social context was critical in shaping how people talked about CSPs. How people drew on the different discourses, and their influences on CSPs, is summarised in Figure 1.

The discourses in support of CSPs were reproduced among young people primarily by their peers and the media and only indirectly by the way parents communicated about CSPs. The discourses discouraging CSPs and portraying them as “harmful” and socially degrading were primarily reproduced by parents, religious leaders and institutions such as schools and health facilities and only occasionally by young people.

Parents blamed new entertainments such as discos, movies and mobile phones as responsible for the transmission of discourses encouraging CSPs. They said these made it difficult for parents to communicate with their children about appropriate values including those discouraging CSPs:

Currently there are many influences such as the media, peers. Apart from these, there is also the issue of going to discos....they just do what they want. (IDI, single mother)

Parents talked about how some discourses supporting CSPs among men were deeply rooted in their culture and passed on subconsciously from one generation to another.

A father said:

We found these issues concerning Sukuma customs in existence… they are just passed on from one generation to another…they were started by our ancestors in the past… we are just passing them on…in the past they were marrying even six wives in one home and the man would live with them all at home. (IDI, father, two parent family)

Parents expressed their disappointment about the influence of peers in their daughters’ decisions to engage in CSPs. A mother said:

Mm, according to the situation we are in with our own children, the situation is completely hard, they have turned themselves into “a meal for men”…what can we do?…when they walk about in the streets/area, they hear, they understand and they apply it in their lives…when girls meet, they discuss, “you know that man, he bought this and that for me”, and that is how they take in the point…Mm, they learn there on the streets. (IDI, mother from two parent family)

## Discussion and implications for HIV prevention

Our findings have delineated six discourses available to parents and young people in talking about CSPs. Two discourses, those of feminine and masculine respectability, had contradictory influences in that they contained elements that both encouraged and discouraged CSPs. Discourses of predatory masculinity and the modern, empowered woman encouraged CSPs while the traditional health beliefs and public health discourses discouraged CSPs.

The predatory masculinity discourse enhances men’s power over females in heterosexual relationship as men feel more masculine the more women that they are able to seduce. This discourse was very much driven by the beliefs that males are the ones in control of relationships and that having CSPs was a proof of one’s manhood. Hence, when young men were with their peers they drew more on the predatory masculinity discourse than on the men’s respectability and public health discourses. This confirms other authors’ observations that masculinity is a key driver of men’s sexual interaction with women [25,33-35].

While young women talked about the value of feminine respectability with regards to social reputation and their future marriageability, when with their peers they positioned themselves as subjects with agency using the discourse of empowered modern young women. Nevertheless, they also used the public health discourse in showing their awareness of the risks of engaging in CSPs and that CSPs were bad. When mothers were with daughters, their discussions drew a lot on the discourses of feminine respectability, traditional beliefs and public health.

The discourses reflect the tensions that exist in how people regard sexual relationships. It is important to understand how at a specific moment several co-existing and potentially contradictory discourses concerning sexuality make available different positions and different powers for men and women. The fact that young people take different positions in different discourses at different times with different people attests the complexity inherent in the meanings people attach to sexuality and how they view themselves. This different positioning has important implications for SRH and HIV prevention interventions, in particular messages in HIV educational materials.

The empowered modern women discourse contradicts the feminine respectability discourse and tends to challenge the predatory masculinity discourse that placed men at the helm of most heterosexual encounters. Young women’s embracement of this discourse points to the changing socio-economic landscape of relationships in these communities [36,37] that seems to be changing young women’s outlook towards their expected traditional positions in sexual relationships. Although ‘empowering’ to young women, or rather creating a feeling of ‘being empowered’, it increases women’s risk of HIV. Other researchers have similarly found that young women feel empowered in sexual relationships because they could determine the amount of sexual exchange [38-40]. The norms in support of the empowered modern women discourse need to be discussed with young women in the light of the risks of CSPs. Sexual health promotion might appropriate and modify this discourse by endorsing young women’s search for empowerment but showing that their agency is best demonstrated by practising safer sex, which puts their health interests above the sexual pleasure of their partners. It might also be possible to engage young women in critical reflection about what being ‘truly empowered’ means with regards to the number of partners one has at a given time.

The feminine respectability discourse emphasised female monogamy, maintaining long term relationships and family life as critical qualities for women. Women’s engagement in CSPs was discouraged through this discourse, although mothers’ advice that their daughters should tolerate men’s engagement in CSPs helped perpetuate the practice. While the predatory masculinity discourse legitimated men’s engagement in CSPs but not women’s, the feminine respectability discourse discouraged women’s premarital sex and condemned their engagement in CSPs. It has been suggested [41] that one of the important strategic ways of addressing women’s and men’s sexual relationships, in particular heterosexual interactions, may be to develop new discourses, or open space for alternative discourses, which contradict and challenge those that are harmful. SRH and empowerment interventions would then focus on altering the feminine respectability, the empowered modern women, masculine respectability and predatory masculinity discourses.

The feminine respectability discourse was responsible for the dichotomization of women as *malaya* [prostitutes] versus ‘nice’ marriageable women. This dichotomy was often alluded to in the predatory masculine sexuality discourse in which men would classify certain women as *malaya,* and so undesirable as long term sexual partners but suitable to demonstrate their sexual appetite and seductive skills. Similar dichotomization of women into ‘nice’ and ‘bad/disreputable’ has been noted by many other authors and is near universal [27,42,43]. Such dichotomization stigmatises women by presenting them as responsible for the spread of HIV. Haram [44] noted the importance of feminine respectability in Northern Tanzania in manoeuvring sexual relationships. According to the culturally prescribed notion of proper conduct young women were more restricted both morally and spatially in managing premarital love affairs compared to men.

It is worth noting the contradictions that existed between the discourses across and within the genders, as other authors have done [43]. The meanings of sex can be contradictory and the liberating effects of a discourse for one group may have contrary implications for another group. The discourse of feminine respectability, for example, encouraged male engagement in CSPs through mothers’ advice to daughters to tolerate men’s polygamous nature, but discouraged female engagement in CSPs through young people’s and parents’ notions of respectable women. Conversely the predatory discourse enhanced men’s rights to heterosexual sex without emotional bonds. Some of the contradictions inherent in the discourses may be difficult to address, but being aware of them allows health promotion to endorse the beneficial elements of a current discourse and try to undermine or modify the negative elements.

Although intergenerational sex was very much in line with the predatory masculinity discourse, it was contrary to the men’s respectability, traditional health beliefs and public health discourses which in most cases discouraged CSPs. Sex between older men and younger women is common in sub-Saharan Africa [34,38,45,46] and understanding discourses around predatory masculinity is a key step in addressing the behaviour that puts many young women at risk of HIV infection from older men.

The discourses of feminine and masculine respectability, predatory masculinity, and traditional health beliefs originated in Sukuma culture, were passed on from one generation to another through socialisation, and were widely endorsed. Since they are deep rooted they may require more careful thought to modify them than the more contemporary discourses. The empowered modern women discourse seems to have emerged in response to social economic changes which have brought new demands for a modern lifestyle for young women. We therefore argue that as young women embrace modernity, they may easily discard traditional ideals that are discouraging CSPs (e.g. feminine respectability) and adopt more trendy ones that give them more agency and are supportive of their sexual behaviours.

Although young people’s sexual behaviour in high income countries may differ from that of young people in low income countries, some of their discourses seem similar. The predatory masculinity discourse identified here corresponds closely with that described by Hollway [25] and Wight [27] under slightly different names - predatory and permissive discourses - while discourses of feminine sexual respectability are almost universal.

A limitation of this analysis is that it does not clarify how discourses relate to practice. It is, therefore, difficult to establish whether the discourses used in the interviews to describe particular behaviours were the same as those through which the behaviours were understood at the time. More fundamental, however, is the difficulty in clarifying whether the discourse within which someone located her/himself prompted certain actions or whether having acted in a particular way, the person adopted a particular discourse through which to interpret their actions [27]. Bryman [47] noted that a person’s discourse is affected by the context that he or she is confronting. In our study, peer groups made certain discourse positions more available and legitimate than others. Thus, we acknowledge that participants’ explanations for engaging in CSPs may vary according to whether they are addressing an interviewer in a research setting, peers, potential partners or family members. Our PO was not extensive enough to resolve these issues.

### Implications for HIV prevention

Understanding how prevalent discourses shape patterns of sexual relationships, and using them as resources in HIV prevention, could contribute to “culturally compelling” interventions to modify CSP practices [48]. This could be done by exploring ways of reinforcing discourses that promote SRH and HIV prevention and modifying those that do not. Although the public health discourse was etic, both parents and young people drew heavily on it when discussing the risks of CSPs, in particular HIV. However, the knowledge transmitted through this discourse clearly was not sufficient to prevent CSPs, perpetuated as they were by other widely prevalent discourses. Rather than attempt to eradicate the empowered modern women discourse, given that it was widely prevalent amongst young women, it might be possible to modify it by using some elements of the discourse to counter other elements. For instance, selecting safer sexual partners, negotiating safer sex, and/or condom use might be presented as demonstrations of modernity, empowerment, autonomy or entrepreneurial acumen through radio advertising, edutainment and popular music. The predatory masculine sexuality discourse offers less scope for adaptation and it might need to be countered by discourses that discourage risk taking.

## Conclusion

Although many participants were aware of the consequences of CSPs, as evident in their use of the public health discourse, they were still motivated to engage in the practice for several conflicting reasons. It is apparent that the relationship between discourses and behaviour is complex but the discourses through which young people and parents discussed CSPs, and the positions people adopted within these discourses, were critical in perpetuating or minimising the practice of CSPs. A better understanding of the discourses young people use to refer to CSPs, and how these discourses are transmitted across generations, might enable interventions to modify these discourses to enhance sexual health in “culturally compelling” ways.

## Competing interests

The authors declare that they have no competing interests.

## Authors’ contributions

JW designed the study, conducted literature searches, led the fieldwork, conducted the analysis, and wrote the first draft of the paper. DW conceived the study and got it funded, provided technical support on all aspects of the study and contributed to successive drafts of this paper. Both authors read and approved the final manuscript.

## Pre-publication history

The pre-publication history for this paper can be accessed here:

http://www.biomedcentral.com/1471-2458/14/742/prepub
